# A Systems-Level Approach for Investigating *Pseudomonas aeruginosa* Biofilm Formation

**DOI:** 10.1371/journal.pone.0057050

**Published:** 2013-02-22

**Authors:** Zhaobin Xu, Xin Fang, Thomas K. Wood, Zuyi Jacky Huang

**Affiliations:** 1 Department of Chemical Engineering, Villanova University, Villanova, Pennsylvania, United States of America; 2 The Henry M. Jackson Foundation for the Advancement of Military Medicine, Bethesda, Maryland, United States of America; 3 Departments of Chemical Engineering and Biochemistry and Molecular Biology, Pennsylvania State University, University Park, Pennsylvania, United States of America; 4 The Center for Nonlinear Dynamics & Control, Villanova University, Villanova, Pennsylvania, United States of America; 5 Villanova Center for the Advancement of Sustainability in Engineering (VCASE), Villanova University, Villanova, Pennsylvania, United States of America; Institut Pasteur, France

## Abstract

Prevention of the initiation of biofilm formation is the most important step for combating biofilm-associated pathogens, as the ability of pathogens to resist antibiotics is enhanced 10 to 1000 times once biofilms are formed. Genes essential to bacterial growth in the planktonic state are potential targets to treat biofilm-associated pathogens. However, the biofilm formation capability of strains with mutations in these essential genes must be evaluated, since the pathogen might form a biofilm before it is eliminated. In order to address this issue, this work proposes a systems-level approach to quantifying the biofilm formation capability of mutants to determine target genes that are essential for bacterial metabolism in the planktonic state but do not induce biofilm formation in their mutants. The changes of fluxes through the reactions associated with the genes positively related to biofilm formation are used as soft sensors in the flux balance analysis to quantify the trend of biofilm formation upon the mutation of an essential gene. The essential genes whose mutants are predicted not to induce biofilm formation are regarded as gene targets. The proposed approach was applied to identify target genes to treat *Pseudomonas aeruginosa* infections. It is interesting to find that most essential gene mutants exhibit high potential to induce the biofilm formation while most non-essential gene mutants do not. Critically, we identified four essential genes, *lysC*, *cysH*, *adk*, and *galU*, that constitute gene targets to treat *P. aeruginosa*. They have been suggested by existing experimental data as potential drug targets for their crucial role in the survival or virulence of *P. aeruginosa*. It is also interesting to find that *P. aeruginosa* tends to survive the essential-gene mutation treatment by mainly enhancing fluxes through 8 metabolic reactions that regulate acetate metabolism, arginine metabolism, and glutamate metabolism.

## Introduction

Biofilms have been frequently associated with human diseases such as osteomyelitis [1], chronic wound infections [2], [3] and cystic fibrosis [4], as they facilitate the survival of pathogens in hostile environments. It is reported that nearly 65% of all nosocomial infections in the USA are associated with biofilms [5]. When exposed to stress, such as that imposed by antibiotic treatments or limited nutrients, pathogens adhere to each other to form biofilms for the purpose of survival [6]. The development of biofilms generally comprises the following four stages, i.e., the initial attachment of planktonic pathogens to a surface, the accumulation of biofilms through the production of extracellular polysaccharide substance (EPS) that interconnects and transiently immobilizes biofilm cells, the maturation of biofilm architecture, and the dispersal of single cells from the biofilm [7]. The first few stages play a key role in treating the biofilm-associated pathogens, as the ability of pathogens to resist antibiotics is significantly enhanced once they form biofilms [5], [7]. Therefore, significant effort in the biofilm research community has been devoted to the investigation of bacterial metabolism and signaling which are involved in the transition from planktonic growth to biofilm growth [8], [9].

Elucidating the mechanisms of biofilm formation is far from trivial: hundreds of highly interacted molecules such as metabolites, metabolic enzymes, and signaling proteins are involved in regulating this process. Most current research is focused on the experimental investigation of the impact of individual molecules, such as regulators, on biofilm formation [10], [11]. This is insufficient for characterizing the biofilm formation process, as a systems-level characterization of interactions between molecules involved in biofilm formation is required to fully understand biofilm formation mechanisms and thus manipulate the metabolism of microorganisms in biofilms. Genome-scale metabolic modeling has been commonly used for systemically studying microorganism metabolism, as evidenced by its wide application in identifying genes that are essential for the growth of *Escherichia coli* [12], *Staphylococcus aureus* [13], *Mycobacterium tuberculosis* [14], *Acinetobacter baumannii* [15], and *Pseudomonas aeruginosa* [16]. A recent study by Thiele and her coworkers [17] shows the first systems biology approach to identifying candidate drug targets for treating *P. aeruginosa* in biofilms. This approach mainly applies single/double gene inhibition simulations to determine the growth of *P. aeruginosa* in specific microenvironments that imitate microbial communities associated with biofilm formation. However, certain issues have not been addressed in this approach, including the quantification of the trend for mutants to form biofilms, and the identification of metabolic reactions that facilitate biofilm formation in mutants. This forms the motivation of this work, that is, to consider the trend of biofilm formation for mutants in the identification of drug-target genes.

Here, we propose an approach to identifying drug targets against a biofilm-forming pathogen, by identifying genes that satisfy two requirements: 1) these genes are crucial for the growth of the pathogen in the planktonic state, that is, the mutation of any of these genes can eliminate the planktonic pathogen; and 2) the inhibition of the function of these genes does not induce biofilm formation.

In particular, we first use the essential planktonic-growth genes presented in Oberhardt et al., 2008 [16] as the initial set of genes satisfying the first requirement, and further, from the initial set, identify genes also satisfying the second requirement. We perform the search by pinpointing a set of biofilm associated reactions from the genes that are reported to be positively related to *P. aeruginosa* biofilm formation in Müsken et al., 2010 [18], and using the flux changes through these reactions as soft-sensors to quantify the impact of the mutation of each gene from the initial set on biofilm formation. The rationale for the use of biofilm-associated reactions is that a large enhancement of fluxes (i.e., activity levels) through these reactions reflects a large potential for a mutant to form biofilms. Specifically, to obtain the flux changes through biofilm-associated reactions, we simulate the metabolism of a gene mutant via flux balance analysis (FBA) [19], and sample the biofilm-associated reaction fluxes via the artificially-center-hit-and-run method (ACHR), an efficient sampling approach for a linearly constrained space [20]. In addition, metabolic reactions whose fluxes significantly increase in most mutants can be determined and regarded as reactions that facilitate biofilm formation. *P. aeruginosa* is chosen as the reference pathogen in this work, because *P. aeruginosa* is one of the leading causes of nosocomial infections in hospitalized patients and *P. aeruginosa* is resistant to a wide array of antibiotics by forming biofilms during chronic infections [21], [22].

## Results

### An illustrative example

**Figure 1:**
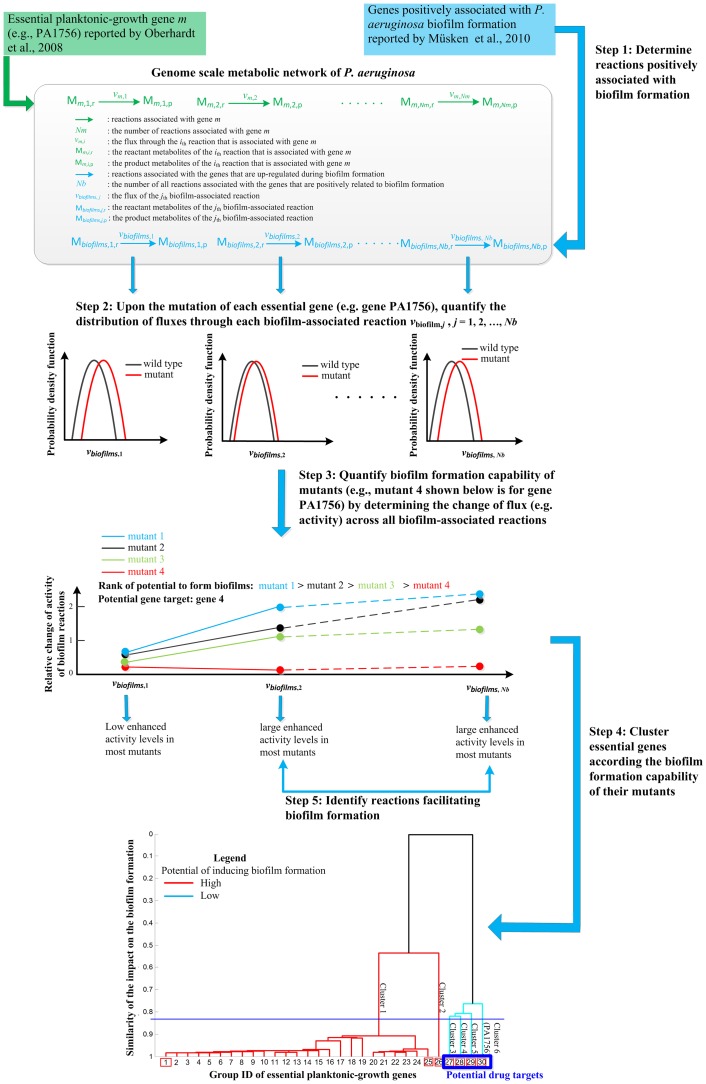
Schematic description of quantifying the biofilm formation capability of single mutants of essential genes for *P. aeruginosa*. The genes positively associated with *P. aeruginosa* biofilm formation are used to determine biofilm-associated reactions in Step 1. The essential gene *m* (e.g., PA1756 gene) is used as a reference gene in Steps 2 through 3 to illustrate the proposed approach for quantifying the potential of a single mutant to form biofilms. Specifically, reactions associated with gene *m* are partially mutated, and the flux distributions of all biofilm-associated reactions for both wide-type and mutant strains are quantified in Step 2. The relative change of activity through all biofilm-associated reactions for the gene *m* mutant is quantified in Step 3. A large enhancement in the activity of biofilm-associated reactions implies a large potential for the single mutant to form biofilms. The profiles for four mutants are given as examples in Step 3. Mutant 4 exhibits the lowest potential to form biofilms. The relative activity change profiles for all single mutants are used to categorize essential genes into different clusters in Step 4. Mutant 4 is assigned to a different cluster from that for the other three mutants, as its relative activity profile is not similar to those for the other mutants in both the shape and the magnitude. The essential planktonic-growth genes whose mutants might not induce biofilm formations are identified from the cluster results and regarded as potential target genes. For example, gene PA1756, which corresponds to mutant 4 in Step 3, is one potential gene target due to the low enhanced activities through those biofilm-associated reactions in its mutant. The biofilm-associated reactions whose activity levels are apparently enhanced in most mutants are identified in Step 5. These reactions indicate the underlying mechanisms for *P. aeruginosa* biofilm formation.

The key innovation of the proposed approach is to take the biofilm formation of a single mutant into account when identifying gene targets to treat biofilm-associated pathogens. While the detail of the proposed approach is given in the Material and Methods section, an example is shown in Figure 1 to illustrate the steps for the identification of drug targets that impair the growth of *P. aeruginosa* without inducing biofilm formation. The genes positively associated with biofilm formation (obtained from Müsken et al., 2010 [18] for *P. aeruginosa* in LB medium) are first overlaid with the *P. aeruginosa* metabolic network to identify reactions associated with biofilm formation (Figure 1, Step 1). The metabolic model presented in Oberhardt et al., 2008 [16], which contains 1056 genes and 883 metabolic reactions, is used in this work, as it is the most comprehensive metabolic model for *P. aeruginosa* and it has been used to correctly predict catabolism of various substrates such as amino acids and common sugars via flux balance analysis. Further, we quantitatively evaluated the impact of the mutation of each essential planktonic-growth gene (obtained from Oberhardt et al., 2008 [16] for *P. aeruginosa* in LB medium, including the PA1756 gene) on biofilm associated reactions, by calculating relative activity changes of these reactions via Steps 2 and 3. The obtained relative activity change profile quantitatively suggests the capability of a mutant to form biofilms, that is, large values for the changes imply a potential induction of the mutant's biofilm formation. In Step 4, the similarity in the shape and magnitude of relative activity profiles is used to categorize the essential planktonic-growth genes [16] into different clusters. The clusters of essential planktonic-growth genes whose mutants have low potential to induce biofilm formation are identified as drug-target genes (such as PA1756), because the mutation of these genes can eliminate planktonic pathogens. Metabolic reactions whose activity levels significantly increase in most single mutants are identified in Step 5. These reactions illustrate how *P. aeruginosa* adjusts its metabolism to form biofilms upon the mutation of genes essential for planktonic growth

### Identification of genes and reactions positively associated with *P. aeruginos*a biofilm formation

A set of biofilm-associated metabolic reactions are obtained via Step 1 in Figure 1. The experimental data of genetic determinants of *P. aeruginosa* biofilm have been presented in Müsken et al., 2010 [18]. 394 genes are reported to be positively associated with biofilm formation, and 64 of them are involved in the metabolic network. None of these 64 genes are essential for bacterial growth in the planktonic state. Flux balance analysis showed that inhibition of 37 of these 64 genes doesn't affect bacterial growth rate. These genes are thus not directly involved in the bacterial biomass synthesis. Fluxes of reactions associated with these 37 genes are the ideal virtual sensors that monitor the trend of *P. aeruginosa* to form biofilms, as these genes are only associated with biofilm formation. The 39 reactions associated with these 37 genes are considered as biofilm-associated reactions (see Table 1).

**Table 1:** Biofilm-associated genes and reactions that are identified via the overlay of the genes reported by Müsken, et al., 2010 [18] to be positively associated with biofilm formation onto the metabolic network presented by Oberhardt, et al., 2008 [16].

Reactions	Genes	Biological subsystems
**Rxn#1**: dGTP + H_2_O → Deoxyguanosine + Inorganic triphosphate	PA1124 (*dgt*)	pyrimidine metabolism
**Rxn#2**: GTP + H_2_O → Guanosine + Inorganic triphosphate	PA1124 (*dgt*)	pyrimidine metabolism
**Rxn#3**: H+ + Malonate → Acetate + CO_2_	PA0208 (*mdcA*)	carbon dioxide production
**Rxn#4**: Coenzyme A + 3-Oxoadipyl-CoA → Acetyl-CoA + Succinyl-CoA	PA0228 (*pcaF*)	acetate metabolism
**Rxn#5**: 4-Aminobutanoate + 2-Oxoglutarate → L-Glutamate + Succinic semialdehyde	PA0266 (*gabT*)	glutamate production
**Rxn#6**: 2 S-Adenosyl-L-methionine + Uroporphyrinogen III → 2 S-Adenosyl-L-homocysteine + H+ + Dihydrosirohydrochlorin	PA0510 (*nirE*)	coenzyme B12 synthesis
**Rxn#7**: Ferrocytochrome c + 2 H+ + Nitrite → Ferricytochrome c + H_2_O + Nitric oxide	PA0511 (*nirJ*)	nitrite consumption
**Rxn#8**: Fumarate + H_2_O ↔ L-Malate	PA0854 (*fumC2*)	TCA cycle
**Rxn#9**: cobalt_2_[e] → cobalt_2_[c]	PA0913 (*mgtE*)	cobalt transport
**Rxn#10**: mg2[e] → mg2[c]	PA0913 (*mgtE*)	magnesium transport
**Rxn#11**: 1-Aminopropan-2-ol + Adenosyl-cobyric acid ↔ Adenosyl cobinamide + H_2_O	PA1275 (*cobD*)	coenzyme B12 synthesis
**Rxn#12**: L-Threonine → 2-Oxobutanoate + Ammonium	PA1326 (*ilvA2*)	production of ammonia
**Rxn#13**: 4-Maleylacetoacetate → 4-Fumarylacetoacetate	PA2007 (*maiA*)	acetate metabolism
**Rxn#14**: Benzoate + 2 H+ + Nicotinamide adenine dinucleotide - reduced + O_2_ → Cis-1,2-dihydroxycyclohexa-3,5-diene-1-carboxylate + Nicotinamide adenine dinucleotide	PA2518 (*xylX*)	benzoate degradation
**Rxn#15**: Isocitrate + Nicotinamide adenine dinucleotide phosphate ↔ 2-Oxoglutarate + CO_2_ + Nicotinamide adenine dinucleotide phosphate - reduced	PA2623 (*icd*)	TCA and carbon dioxide production
**Rxn#16**: 3 H+ + Nicotinamide adenine dinucleotide - reduced + Ubiquinone-8 → Nicotinamide adenine dinucleotide + Ubiquinol-8 + 2 H+	PA2642 (*nuoG*)	oxidative phosphorylation
**Rxn#17**: ATP + H_2_O + Phosphonate[e] → ADP + H+ + Phosphate + Phosphonate[c]	PA3383 (*phnD*)	phosphate transport
**Rxn#18**: S-Adenosyl-L-methionine + Butyryl-[acyl-carrier protein] → 5-Methylthioadenosine + Acyl carrier protein + H+ + N-butyryl-L-homoserine lactone	PA3476 (*rhlI*)	homoserine synthesis
**Rxn#19**: 2 Ferrocytochrome c + 4 H+ + 0.5 O_2_ → 2 Ferricytochrome c + H_2_O + 2 H+	PA4133 (*ccoN*)	iron metabolism
**Rxn#20**: ATP + H_2_O + Fe-enterobactin → ADP + Fe-enterobactin + H+ + Phosphate	PA4160 (*fepD*)	iron metabolism
**Rxn#21**: Chorismate → Isochorismate	PA4231 (*pchA*)	oxidative phosphorylation
**Rxn#22**: 2 Hydrogen peroxide → 2 H_2_O + O_2_	PA4236 (*katA*)	hydrogen peroxide consumption
**Rxn#23**: Alpha-D-Ribose 5-phosphate + Uracil ↔ H_2_O + Pseudouridine 5′-phosphate	PA4544 (*rluD*)	pyrimidine metabolism
**Rxn#24**: 1.5 O_2_ + Protoporphyrinogen IX → 3 H_2_O + Protoporphyrin	PA4664 (*hemK*)	coenzyme B12 synthesis
**Rxn#25**: Alpha-Oxo-benzeneacetic acid ↔ Benzaldehyde + CO_2_	PA4901 (*mdlC*)	carbon dioxide production
**Rxn#26**: Reduced glutathione + Methylglyoxal → (R)-S-Lactoylglutathione	PA5111 (*gloA3*)	glutathione metabolism
**Rxn#27**: N(omega)-(L-Arginino)succinate ↔ L-Arginine + Fumarate	PA5263 (*argH*)	arginine metabolism
**Rxn#28**: ATP + H_2_O + Phosphate[e] → ADP + H+ + 2 Phosphate[c]	PA5368 (*pstC*)	phosphate transport
**Rxn#29**: Citrate[e] + H+[e] ↔ Citrate[c] + H+[c]	PA5476 (*citA*)	TCA cycle
**Rxn#30**: 5,6-dihydrouracil + H_2_O ↔ N-Carbamoyl-beta-alanine + H+	PA0441 (*dht*)	pyrimidine metabolism
**Rxn#31**: Nicotinamide adenine dinucleotide + O-Phospho-4-hydroxy-L-threonine → 2-Amino-3-oxo-4-phosphonooxybutyrate + H+ + Nicotinamide adenine dinucleotide − reduced	PA0593 (*pdxA*)	pyridoxine metabolism
**Rxn#32**: 2-Methyl-4-amino-5-hydroxymethylpyrimidine diphosphate + 4-Methyl-5-(2-phosphoethyl)-thiazole + H+ → Diphosphate + Thiamin monophosphate	PA3976 (*thiE*)	thiamin metabolism
**Rxn#33**: ATP + Coenzyme A + Succinate ↔ ADP + Phosphate + Succinyl-CoA	PA1588 (*sucC*)	TCA cycle
**Rxn#34**: L-Aspartate + ATP + L-Citrulline → AMP + N(omega)-(L-Arginino)succinate + H+ + Diphosphate	PA3525 (*argG*)	arginine metabolism
**Rxn#35**: Acetate + ATP + Coenzyme A → Acetyl-CoA + AMP + Diphosphate	PA4733 (*acsB*)	acetate metabolism
**Rxn#36**: 2 ATP + L-Glutamine + H_2_O + Bicarbonate → 2 ADP + Carbamoyl phosphate + L-Glutamate + 2 H+ + Phosphate	PA4758 (*carA*)	arginine metabolism
**Rxn#37**: 2 H+ + H_2_O + Urea → CO_2_ + 2 Ammonium	PA4867 (*ureB*)	arginine metabolism, ammonia production, carbon dioxide production
**Rxn#38**: H_2_O + Urocanate → 4-Imidazolone-5-propanoate	PA5100 (*hutU*)	glutamate metabolism
**Rxn#39**: ATP + Oxaloacetate → ADP + CO_2_ + Phosphoenolpyruvate	PA5192 (*pckA*)	carbon dioxide production

It can be seen from Table 1 that the biofilm-associated reactions are mainly involved in the consumption of nitrite, the regulation of acetate, tricarboxylic acid (TCA) circle, the generation of carbon dioxide and ammonia, iron metabolism, the regulation of hydrogen peroxide, arginine metabolism, the production of glutamate, pyrimidine metabolism, and oxidative phosphorylation. While these reactions were identified by overlaying the 37 genes that are reported to be associated with biofilm formation in Müsken et al., 2010 [18] onto the metabolic network of *P. aeruginosa*, their important role in biofilm formation is further confirmed by the following independent evidence:

- It has been shown that accumulation of nitrite is required to inhibit biofilm formation in staphylococcal strains [23]. The consumption of nitrite thus facilitates biofilm formation.
- Acetate is found to be engaged in the switch from a physiology program that permits a rapid growth in the presence of abundant nutrients to a program related to biofilm formation that can enhance the survival of *E. coli* in the absence of nutrients [24].
- Certain genes in TCA cycle are up-regulated in the staphylococcal biofilm [25].
- The concentration of carbon dioxide can be used as an indicator of biofilm formation [26], and carbon dioxide and ammonia influence the pH value in the cytosol, which in turn affect biofilm formation [18].
- The important role of iron in the *E. coli* biofilm formation has been reported by several previous studies [27], [28], [29].
- Hydrogen peroxide has been recognized to trigger the initiation of biofilm in *Streptococcus gordonii* via the release of the extracellular DNA [30].
- The arginine fermentation regulated by the arginine deaminase is the major metabolic process for the generation of ATP in *P. aeruginosa* in the anaerobic condition [31].
- Glutamate synthesis is reported to be positively correlated with biofilm formation of *Mycobacterium bovis* [32].
- Pyrimidine metabolism is reported to participate in the modulation of the *E. coli* biofilm formation [33]. It has been experimentally verified that pyrimidine uracil influences quorum sensing and biofilm formation in *P. aeruginosa* [34].
- Oxidative phosphorylation is coupled with the ATP production for pathogens in biofilms. It has been demonstrated that enzymes that participate in the oxidative phosphorylation are up-regulated in *Bordetella pertussis* biofilms [35].

### Investigation of biofilm formation capability for single-mutants

**Figure 2:**
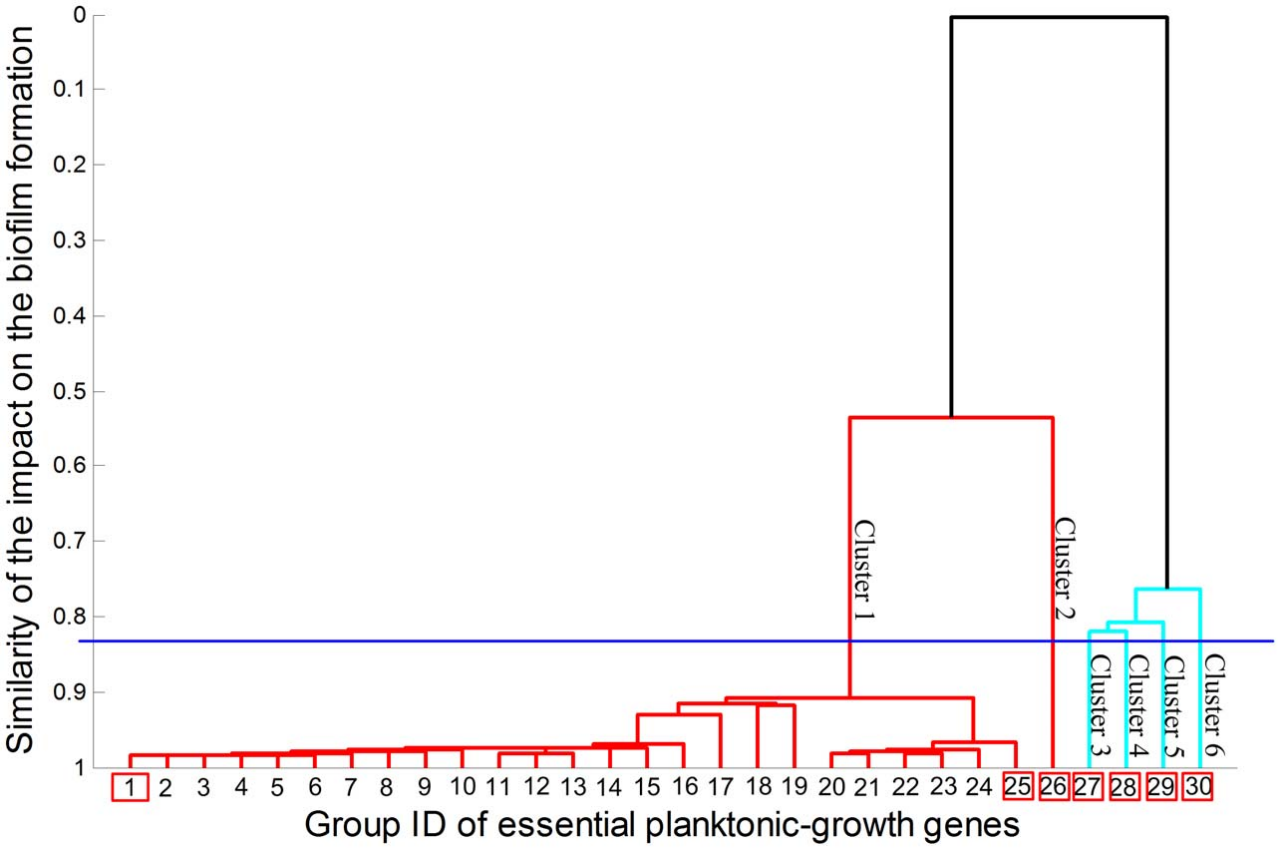
Clustering result for essential planktonic-growth genes based upon the ability of their mutants to form biofilms. These essential genes are separated into 30 groups by the hierarchical clustering program. The genes in each group are listed in Table 2. They are categorized into six clusters by selecting a threshold marked by the blue color line. The groups marked by red rectangles are regarded as the representatives of the cluster of genes. Two groups with the lowest similarity in the same cluster are selected as representatives if more than one group of genes are involved in that cluster.

Given the above biofilm-associated reactions, we obtained a relative activity change profile of these reactions for each single mutant of the 136 essential planktonic-growth genes obtained from Oberhardt et al., 2008 [16] (via Steps 2 and 3), and, subsequently, based upon the similarity in relative activity change profiles for different single mutants, went through Step 4 to hierarchically categorize the essential planktonic-growth genes into six different clusters (as shown in Figure 2). Clusters 1 and 2 are located in one branch, while the other four clusters are associated with the other branch. The hierarchical clustering algorithm allows us to further separate genes in each cluster into different groups according to the similarity in their relative activity change profiles. The genes associated with each group shown in Figure 2 are listed in Table 2.

**Table 2:** Genes associated with each group shown in Figure 2.

Group ID	Cluster	# of genes in each group	Essential planktonic-growth genes
1	1	6	**PA0945(** ***purM*)**,PA4693(*pssA*),PA4855(*purD*),PA4957(*psd*), PA5164(*rmlC*), PA5549(*glmS*)
2	1	1	PA5161(*rmlB*)
3	1	1	PA3639(*accA*)
4	1	99	PA0005(*lptA*),PA0006(*yaeD*),PA0280(*cysA*),PA0281(*cysW*), PA0282(*cysT*),PA0283(*sbp*),PA0342(*thyA*),PA0350(*folA*), PA0363(*coaD*),PA0430(*metF*),PA0582(*folB*),PA0724(*coaA*), PA0761(*nadB*),PA1004(*nadA*),PA1013(*purC*),PA1162(*dapE*), PA1376(*aceK*),PA1393(*cysC*),PA1493(*cysP*),PA1758(*pabB*), PA1796(*folD*),PA1806(*fabI*),PA1959(*bacA*),PA2165(*glgA*), PA2584(*pgsA*),PA2629(*purB*),PA2962(*tmk*),PA2964(*pabC*), PA2967(*fabG*),PA2977(*murB*),PA2979(*kdsB*),PA2981(*lpxK*), PA3088(*yfjB*),PA3108(*purF*),PA3111(*folC*),PA3112(*accD*), PA3163(*cmk*),PA3337(*rfaD*),PA3636(*kdsA*),PA3637(*pyrG*), PA3643(*lpxB*),PA3644(*lpxA*),PA3651(*cdsA*),PA3666(*dapD*), PA3673(*plsB*),PA4006(*nadD*),PA4050(*pgpA*),PA4053(*ribE*), PA4054(*ribB*),PA4055(*ribC*),PA4056(*ribD*),PA4201(*ddlA*), PA4397(*panE*),PA4406(*lpxC*),PA4410(*ddlB*),PA4411(*murC*), PA4412(*murG*),PA4414(*murD*),PA4415(*mraY*),PA4416(*murF*)PA4417(*murE*),PA4425(*yraO*),PA4442(*cysN*),PA4443(*cysD*), PA4450(*murA*),PA4457(*KdsD*),PA4524(*nadC*),PA4529(*coaE*), PA4561(*ribF*),PA4655(*hemH*),PA4666(*hemA*),PA4670(*prs*), PA4729(*panB*),PA4730(*panC*),PA4749(*glmM*),PA4750(*folP*), PA4759(*dapB*),PA4847(*accB*),PA4848(*accC*),PA4854(*purH*), PA4920(*nadE*),PA4938(*purA*),PA4988(*waaA*),PA4996(*rfaE*), PA5009(*waaP*),PA5010(*waaG*),PA5011(*waaC*),PA5012(*waaF*)PA5034(*hemE*),PA5162(*rmlD*),PA5163(*rmlA*),PA5175(*cysQ*), PA5243(*hemB*),PA5259(*hemD*),PA5260(*hemC*),PA5278(*dapF*)PA5320(*coaC*), PA5336(*gmk*), PA5552(*glmU*)
5	1	2	PA3646(*lpxD*),PA3807(*ndk*)
6	1	1	PA3654(*pyrH*)
7	1	1	PA2968(*fabD*)
8	1	1	PA0654(*speD*)
9	1	1	PA1614(*gpsA*)
10	1	1	PA1609(*fabB*)
11	1	1	PA3763(*purL*)
12	1	1	PA4662(*murI*)
13	1	2	PA4458(*yrbI*), PA5008(*waaX*),
14	1	1	PA3659(*dapC*)
15	1	1	PA1687(*speE*)
16	1	1	PA3296(*phoA*)
17	1	1	PA0546(*metK*)
18	1	1	PA2053(*cynT*)
19	1	1	PA4748(*tpiA*)
20	1	2	PA0025(*aroE*),PA5039(*aroK*)
21	1	1	PA1681(*aroC*)
22	1	1	PA0548(*tktA*)
23	1	1	PA3164(*pseudogene*)
24	1	1	PA0330(*rpiA*)
25	1	1	**PA5038(** ***aroB*)**
26	2	1	**PA4031(** ***ppa*)**
27	3	1	**PA0904(** ***lysC*)**
28	4	1	**PA2023(** ***galU*)**
29	5	1	**PA3686(** ***adk*)**
30	6	1	**PA1756(** ***cysH*)**

The representative genes are underlined and marked in bold.

**Figure 3:**
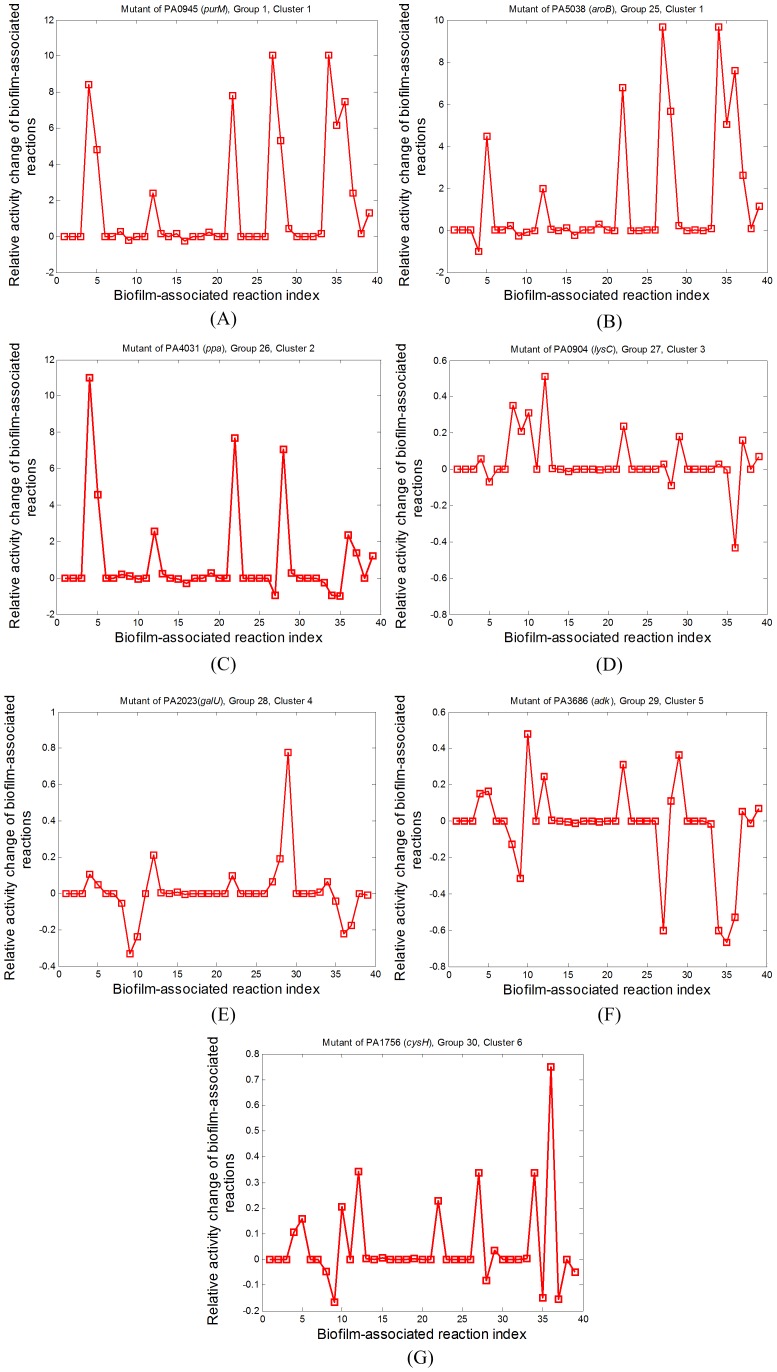
The relative activity change of biofilm-associated reactions for representative mutants. (A) PA0945 from Group 1 in Cluster 1, (B) PA5038 from Group 25 in Cluster 1, (C) PA4031 from Group 26 in Cluster 2, (D) PA0904 from Group 27 in Cluster 3, (E) PA2023 from Group 28 in Cluster 4, (F) PA3686 from Group 29 in Cluster 5, and (G) PA1756 from Group 30 in Cluster 6. The relative activity change of a biofilm-associated reaction is quantified by the relative flux change of this reaction upon the mutation of a single essential gene. The biofilm formation capability of a mutant is indicated by the relative activity change across all biofilm-associated reactions. If certain biofilm-associated reactions exhibit significantly enhanced activity levels, the mutant has large potential to form biofilms. Approximately 10 of the biofilm-associated reactions are of significantly increased activity levels upon the mutation of the genes from Cluster 1, and the activity levels of 6 biofilm-associated reactions surge in the mutants of the genes from Cluster 2. The mutants from Clusters 1 and 2 thus have high potential to form biofilms. Compared to the large relative activity increases of biofilm-associated reactions for mutants associated with Clusters 1 and 2, the activity level increases are minor for the mutants of the genes from Clusters 3 through 6. These mutants thus have low potential to form biofilms.

The profiles of relative activity change for the mutants of the representative groups for different clusters are shown in Figure 3. Figure 3 (A) and (B) show the profiles for the mutants of the PA0945 (from Group 1, Cluster 1) and PA5038 genes (from Group 25, Cluster 1), respectively. The profiles of these two mutants are used to represent the whole spectrum of Cluster 1, because, although they are from the two groups (Groups 1 and 25) with the lowest similarity in the cluster, their profiles of relative activity change in Figure 3 (A) and (B) are similar, suggesting that the single mutations of genes in Cluster 1 have similar impacts on biofilm-associated reactions and thus potentially on biofilm formation. Figure 3 (C) through (G) show the relative activity change profiles of representative groups from Clusters 2 through 6, respectively. Since there is only one group (actually one gene per group) associated with each of these clusters, these groups are regarded as the representatives of the corresponding clusters (as shown in Figure 2).

Single mutants of genes from different clusters have different potentials to induce biofilm formation. As shown in Figure 3 (A) and (B), the activity levels of certain biofilm-associated reactions have significant increases upon the mutation of a gene from Cluster 1, suggesting that the gene mutants from Cluster 1 might form biofilms and in turn prevent the elimination of *P. aeruginosa*. The comparison of Figure 3 (C) to Figure 3 (A) and (B) shows that mutants from Cluster 2 might still form biofilms but to a lesser extent. In particular, the mutant from Cluster 2 (PA4031) and those from Cluster 1 (PA0945 and PA5038) have similar relative activity changes in the biofilm-associated reactions Rxn#1 through Rxn#32, but the mutant from Cluster 2 has much smaller changes in Rxn#33 through Rxn#39 than those from Cluster 1. Interestingly, as shown in Table 2, there are totally 132 essential genes associated with Cluster 1 and Cluster 2, while only the remaining four are from Clusters 3 through 6, suggesting that the mutation of most essential planktonic-growth genes might induce the formation of biofilms. Therefore, not every essential planktonic-growth gene is a good target to treat biofilm-associated pathogens.

The mutation of any gene from Clusters 3 through 6 might not induce biofilm formation, as approximately 96% of the biofilm-associated reactions for these mutants are of small relative activity changes (e.g. most less than 0.5). In addition, the relative activity changes of certain biofilm-associated reactions are reversed and have negative values. Even though the amplitudes of these negative relative fold changes are minor (e.g., most less than 0.5), the metabolic activities are not distributed in the direction for promoting biofilm formation for the mutants from Clusters 3 through 6. Therefore, the four genes in these clusters, i.e., PA0904 (*lysC*), PA1756 (*cysH*), PA3686 (*adk*), and PA2023(*galU*), are regarded potential gene targets to treat *P. aeruginosa*, because they are essential to planktonic *P. aeruginosa* and their mutants might not induce biofilm formation.

### Validation of the predicted results with existing experimental data

Experimental data were collected from the literature to validate the aforementioned prediction results, which shows that the mutants from Clusters 1 and 2 might induce biofilm formation while the mutants from Clusters 3 through 6 might not. The essential genes contained in Clusters 1 and 2 are mainly involved in vitamin and cofactor synthesis, amino acid catabolism, cell wall synthesis, central metabolism, the membrane transport system, nucleotide synthesis, nucleotide salvage, and lipid synthesis. The involvement of these genes in the aforementioned biological systems is listed in the Table S1. The mutants of some genes in Cluster 1 have been reported to form biofilms. For example, the inhibitor of the enzyme encoded by the *glmU* gene (Group 4) is able to enhance the formation of biofilms of *P. aeruginosa* PAO1 [36]. Another example is given that the *pgsA* null mutant (Group 4) of *E. coli* activates the Rcs signal transduction pathway that is crucial for *E. coli* biofilm formation [37]. In addition, the down-regulation of the following six essential genes, i.e., *pyrH* (Group 6), *tktA* (Group 22), *tpiA* (Group 19), *rpiA* (Group 24), *dapD* (Group 4), and *rmlA* (Group 4), is reported as the driving force for *Streptococcus* biofilm formation [38].

As seen from Figure 3, the activity levels of all the biofilm-associated reactions are small for the genes from Clusters 3 through 6, i.e., PA0904 (*lysC*), PA1756 (*cysH*), PA2023(*galU*), and PA3686 (*adk*). Hence, they are regarded as ideal gene targets to treat *P. aeruginosa* infections because the inactivation of any of them can kill the planktonic pathogen cells but cannot promote the switch from planktonic growth to biofilm formation. The following experimental evidence further confirms these four genes are good targets to treat biofilm-associated *P. aeruginosa*:

- The enzyme encoded by *lysC* is aspartate kinase (AK) that catalyzes the phosphorylation of aspartic acid, the first step in the biosynthesis of the aspartic amino-acid family, lysine, threonine, and methionine. Aspartate kinase can also influence the synthesis of diaminopimelic acid, which is an intermediate in the lysine biosynthesis and meanwhile a key compound participating in cell-wall synthesis in most bacteria [39]. Furthermore, it has been found that the mutation of *lysC* is unable to form biofilms in *Vibrio cholerae* [40].
- *cysH* encodes adenosine 5′-phosphosulfate reductase that catalyzes the first committed step of reductive sulfate assimilation in *P. aeruginosa*. This enzyme is required for the survival and virulence of *P. aeruginosa* in biofilms [41].
- Gene *galU* encodes glucose-1-phosphate uridylyltransferase, which mediates the reversible reaction of glucose-1-phosphate (G1P) and UTP into UDP-glucose and pyrophosphate. The function of glucose-1-phosphate uridylyltransferase in microorganisms is to synthesize capsular polysaccharide that is essential for the survival and virulence of *P. aeruginosa* in biofilm [42].
- The enzyme encoded by *adk* is adenylate kinase, a ubiquitous enzyme that contributes to the homeostasis of adenine nucleotides in eukaryotic and prokaryotic cells. It is interesting to find that adenylate kinase functions as a *P. aeruginosa* virulence factor [43]. In particular, adenylate kinase is secreted by *P. aeruginosa*, and it in turn causes the death of human macrophage cell.

### Investigation of metabolic reactions that facilitate biofilm formation

An interesting finding by inspecting Figure 3 (A) through (C) is that certain biofilm-associated reactions have significantly increased activity levels (e.g., more than three-fold) upon the single mutations of all the three representative genes from Cluster 1 and Cluster 2, i.e., PA0945 (*purM*), PA5038 (*aroB*), and PA4031 (*ppa*). This implies that, although these metabolic reactions are all associated with biofilm formation, certain parts of them are more likely to be utilized by *P. aeruginosa* upon the stress such as that imposed by the mutation of essential planktonic growth genes.

**Figure 4:**
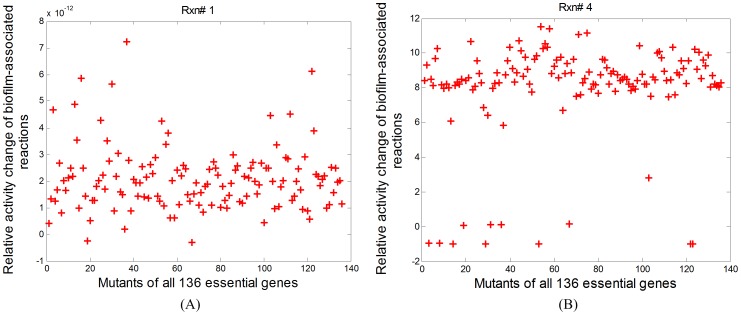
Relative fold change in activity levels of two biofilm-associated reactions for all single mutants. (A) Reaction Rxn# 1, and (B) Reaction Rxn# 4. The activity level of Rxn#1 is of little change for most essential mutants, while the fluxes are significantly re-distributed through Rxn# 4 for most mutants. In other words, Rxn# 4 is of much higher activity levels upon the mutation of most essential genes. This implies that Rxn# 4 stands for the mechanism utilized by mutants to form biofilms. Based upon the relative activity change profiles, the biofilm-associated reactions are categorized into two types, one with minor flux changes upon the mutation of most essential genes (represented by Rxn# 1), and the other with large flux changes upon the mutation of most essential genes (represented by Rxn# 4).

Therefore, we analyzed the relative activity changes of each biofilm-associated reaction across all single mutants, and categorized the 39 biofilm-associated reactions into two types: 1) the reactions with minor increase of activity levels for most essential mutants and 2) those with significant activity increase. Figure 4 (A) and (B) show the typical relative activity changes of two biofilm-associated reactions: a minor-increase reaction (Reaction Rxn# 1) and a “significant-increase” reaction (Reaction Rxn# 4). The relative activity changes of other biofilm-associated reactions are similar to either the one for Rxn# 1 or that for Rxn# 4 (data not shown). Reactions associated with each of the two types of reactions are listed in Table 3.

**Table 3:** Categorization of the biofilm-associated reactions based upon their relative activity changes in the mutants of 136 essential planktonic-growth genes.

Category of biofilm-associated reactions	Biofilm-associated reactions included in each cluster
“Minor-increase”: reactions with minor activity changes for most 136 single mutants	**Rxn#1∼3, 6∼21, 23∼26, 29∼33, 37∼39 in ** **Table 1**
“Significant-increase”: reactions with large increase (e.g., more than three-fold relative change) in their activity levels for most single mutants	**Rxn#4**: Coenzyme A + 3-Oxoadipyl-CoA → Acetyl-CoA + Succinyl-CoA
	**Rxn#5**: 4-Aminobutanoate + 2-Oxoglutarate → L-Glutamate + Succinic semialdehyde
	**Rxn#22**: 2 Hydrogen peroxide → 2 H_2_O + O_2_
	**Rxn#27**: N(omega)-(L-Arginino)succinate ↔ L-Arginine + Fumarate
	**Rxn#28**: ATP + H2O + Phosphate[e] → ADP + H+ + 2 Phosphate[c]
	**Rxn#34**: L-Aspartate + ATP + L-Citrulline → AMP + N(omega)-(L-Arginino)succinate + H+ + Diphosphate
	**Rxn#35**: Acetate + ATP + Coenzyme A → Acetyl-CoA + AMP + Diphosphate
	**Rxn#36**: 2 ATP + L-Glutamine + H_2_O + Bicarbonate → 2 ADP + Carbamoyl phosphate + L-Glutamate + 2 H+ + Phosphate

The “significant-increase” type of reactions have significant increases in activity levels for the mutants of most essential genes, and might reveal the underlying mechanisms for biofilm formation when *P. aeruginosa* is treated by the antimicrobial agents that attack the essential genes. It can be seen from Table 3 that these reactions are mainly involved in acetate metabolism (mainly via Rxn# 4 and 35), arginine metabolism (mainly via Rxn#27 and 34), glutamate metabolism (mainly via Rxn# 5 and 36), the regulation of hydrogen peroxide (mainly via Rxn# 22), and the phosphate transport (mainly via Reaction Rxn #28). These findings are supported by existing experimental data listed as follows.

- The microarray data presented in Prüss et al, 2010 [44] show that acetate metabolism acts as a metabolic sensor for adjusting the biofilm structure of *E. coli* K12 to the change of the surrounding environment.
- It is reported by Beenken et al., 2004 [45], that the arginine plays an important role in generation of ammonia that can neutralize acids generated by bacterial glycolysis in *Staphylococcus aureus* biofilms.
- The isomerization process of glutamate is one of the crucial steps influencing the transport and accumulation of extracellular substances for biofilm formation of *Bacillus amyloliquefaciens* [46].
- Hydrogen peroxide is involved in the lysine oxidase activity that causes cell death within micro-colonies during biofilm formation of both *Marinomonas mediterranea* and *Pseudoalteromonas tunicata* [47].
- It is reported by Monds et al., 2007 [48], that the extracellular phosphate in the environmental conditions is involved in the regulation of *Pseudomonas fluorescens* Pf0-1 biofilm formation. The phosphate transport thus influences the biofilm development by coordinating the extra and intracellular phosphate concentrations.

While all the aforementioned experiments focus on the investigation of the impact of individual metabolic modules on biofilm formation, this work represents the first mathematical modeling approach for the systemic identification of the underlying metabolic mechanisms that facilitate biofilm formation of single-mutants.

## Discussion

The formation of biofilms facilitates the survival of disease-causing pathogens in hostile environmental conditions. Therefore, preventing the pathogen's transition from the planktonic state to the biofilm growth mode is one of the most important steps to treat biofilm-associated pathogens. Since the metabolism of pathogens is determined by the interaction of hundreds of metabolic reactions, genes, and enzymes, systems biology approaches can facilitate gene target identification for preventing the planktonic to biofilms transition. In this work, a systems-level analysis approach was presented to answer the following question that still remains unanswered, that is, how to mathematically quantify the capability of a pathogen to form biofilms upon the mutation of a specific gene. The fluxes through the reactions associated with an essential planktonic-growth gene are limited to 10% of their nominal values in flux balance analysis to mimic the mutation of the gene in this work. Although the approach used here is a partial shutdown instead of a complete gene-knockout mutation, it better reflects the response of pathogens to the treatment of antimicrobial agents, which generally cannot immediately eliminate the biological functions of the target genes. The mutant might form biofilm before antimicrobial agents completely eliminate the bacterial metabolism. In addition, the pathogen might be treated by a sub-inhibition dose, which can be mimicked by setting the allowable fluxes to 10% of their nominal values. The terminology “mutant” or “mutation” is still used in this work, although an in silico partial shutdown mutation is performed to identify gene targets from those essential planktonic-growth genes of *P. aeruginosa.*

Based upon the results in this work, it is interesting to hypothesize that the mutations of essential genes are more likely to induce *P. aeruginosa* biofilm than those of non-essential ones. For essential genes, the mutations of 132 out of the 136 essential planktonic-growth genes were predicted to induce biofilm formation, and the down-regulation of 8 of them, i.e., *pyrH*, *tktA*, *tpiA*, *rpiA*, *dapD*, and *rmlA* [38], *glmU* [36], and *pgsA* [37], has been experimentally proven to be positively correlated with biofilm formation. In contrast, we find that only two out of 920 non-essential mutants might induce biofilm formation, when applying the proposed approach to evaluate the biofilm formation capability of mutants of the nonessential genes in the *P. aeruginosa* model reported by Oberhardt et al., 2008 [16]. For the 920 non-essential genes, we have followed the experiment approaches presented in Ueda et al., 2009 [34], to carry out comprehensive screening experiment for altered biofilm mutants from the PA14 non-redundant transposition mutant library (Liberati et al., 2006, [49]). The library contains 835 of the 920 non-essential genes, and of these 835 mutants, 823 have been verified not to induce biofilm formation (data can be provided upon request). While we predicted a lower number of mutants that form biofilms (i.e., two by prediction versus 12 by experiment), this is probably because we use a conservative approach in mimicking partial shutdown mutation, in which a reaction that is catalyzed by multiple enzymes is not inhibited for single mutants, but on the whole the results were verified for the vast majority of the bacteria with mutations in non-essential genes. The potential reason for explaining the hypothesized strong biofilm formation capability of most essential mutants is that the mutation of an essential planktonic-growth gene might cause limited nutrient uptakes, which strongly enhance biofilm formation [6], and make the pathogen feel the stress, which is reported as one of the major driving forces for biofilm formation [50]. On the contrary, when a non-essential gene is inhibited, the pathogen does not feel very much stress, because non-essential genes are not crucial for the biomass synthesis. This hypothesis as well as the findings of the biofilm formation phenotypes of single mutants forms the foundation for the further experimental investigation.

This work was mainly focused on investigating the biofilm formation capability of single mutants, as the single gene inhibition is easier to implement than the multiple gene inhibition. While it is possible to apply the proposed approach to multiple-mutants, the ACHR sampling approach needs to be upgraded to improve the computational efficiency. It takes approximately 15 minutes to obtain 20,000 samples of fluxes of the biofilm-associated reactions upon the mutation of a single gene for a desktop computer with Intel Core i5 2.5 GHz CPU and 8 GB RAM. Since 920 nonessential genes have been reported in Oberhardt et al., 2008 [16], it is very time-consuming to study the biofilm formation ability of all possible multiple-mutants. Nevertheless, the study of the impact of the multiple-gene mutation on biofilm formation is an interesting topic for the future research.

To the best knowledge of the authors, this work represents the first systems-level approach to incorporate the quantification of biofilm formation capability in evaluation of genes as the targets to treat biofilm-associated pathogens. Four essential planktonic-growth genes, i.e., PA0904 (*lysC*), PA1756 (*cysH*), PA3686 (*adk*), and PA2023(*galU*), were identified as the potential gene targets to treat *P. aeruginosa*, as the mutation of any of these genes can eliminate the pathogen without inducing biofilm formation. This finding is implied by existing experimental data. Based upon the relative activity change of biofilm-associated reactions over all the single mutants, it is interesting to find that the fluxes of approximately 8 biofilm-associated reactions significantly increase for most single essential mutants. They are mainly associated with acetate metabolism, arginine metabolism, and glutamate metabolism. All these findings can be used to generate hypotheses for experiment design. In addition, the proposed approach can be applied to identify gene targets for treating any other biofilm-associated pathogen if the genes positively associated with biofilm formation have been identified and the metabolic model has been developed for the pathogen.

## Materials and Methods

In this section, the systems-level approach whose outline is shown in the illustrative example (Figure 1) is described. This approach is used to quantify the biofilm formation capability of single essential mutants, cluster essential genes according to the biofilm formation capacity of their mutants, and systematically identify the metabolic reactions whose activity levels significantly increase for most single essential mutants. The detail of the proposed approach is shown step by step as follows.

- Step 1: Genes positively associated with biofilm formation of the target pathogen are identified and are overlaid onto the metabolic network of the pathogen to determine the reactions positively associated with biofilm formation.The fluxes of these biofilm-associated reactions are used as soft sensors to monitor the ability of a mutant to form biofilm. In particular, if the fluxes (i.e., activity levels) of certain biofilm-associated reactions significantly increase upon the mutation of an essential planktonic-growth gene, the pathogen might form biofilms before it is eliminated by the antimicrobial agents which attack that essential gene. In other words, the essential gene is not a good target to treat the pathogen.
- Step 2: An integrated flux balance analysis and ACHR sampling approach is applied to determine the relative change of the flux distribution of individual biofilm-associated reactions upon the mutation of an essential gene.FBA is a standard tool to determine the reaction fluxes through the entire metabolic network under the assumption that the microorganism utilizes available nutrients to get the maximum growth rate [19], [51]. FBA can be regarded as a linear programming problem given by Equation (1).
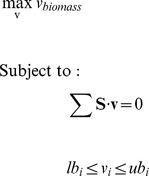
where *v_biomass_* is the biomass growth rate, 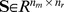
is the stoichiometric matrix, *n_m_* and *n_r_* are the number of metabolites and metabolic reactions respectively, **v** is a vector containing fluxes for all reactions in the metabolic network, *lb_i_* and *ub_i_* are the lower and upper bounds of flux *v_i_*. FBA is performed to predict the maximum microorganism growth rate under a specific nutrient environment specified by the lower and upper bounds of fluxes. Equation (1) can be solved by the program package GNU Linear Programming Kit (GLPK) [52].Since the number of metabolic reactions (i.e., *n_r_*) is generally larger than the number of metabolites (i.e., *n_m_*) in the reaction network, the stoichiometric matrix **S** is underdetermined. The optimal solution for Equation (1) is typically not unique [53]. In this work, the solution space corresponding to the maximum growth rate is sampled using ACHR sampling. In particular, the ACHR sampling routine provided by the Cobra toolbox [19], [51] is used to get *N* flux samples for each metabolic reaction from the solution space, where *N* is a large number (e.g., 20,000) that can make the sampling process converge. For each metabolic reaction, its *N* sampled flux values are analyzed to quantify the mean value (represented by 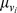), and the probability density function of fluxes (represented by 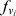) in the solution space. *i* is the index of a metabolic reaction.The aforementioned approach is used to sample fluxes of biofilm-associated reactions for the wild-type strain and the single essential mutants. The corresponding mean value, and probability density function quantified from the sampled fluxes for each biofilm-associated reaction are represented by 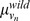 and 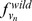 for the wild-type strain, and 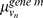 and 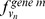 for gene *m* mutant. *n* is the index of a biofilm-associated reaction.To mimic the mutation of an essential gene *m* in flux balance analysis, the upper bounds of fluxes of reactions associated with the essential gene *m* are set to 10% of the mean value of their *N* sampled fluxes obtained for the wild type strain. If a reaction is reversible, the lower bound of its flux is set to −10% of the mean value of its *N* sampled fluxes obtained for the wild type strain. The pathogen might form biofilms before the gene target is totally inhibited. A 90% flux reduction can mimic the growth pattern of the pathogen before antimicrobial agents completely abolish the biological functions of their targets. In addition, the sub-inhibition of a good gene target should not induce the formation of biofilms before antimicrobial agents totally inhibit its biological functions.
- Step 3: The trend to form biofilms for the mutant of essential gene *m* is quantified by comparing the distribution of fluxes of each biofilm-associated reaction, represented by 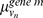 and 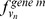, to its counterpart 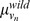 and 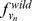 obtained for the wild-type strain.The Kolmogorov – Smirnov (K-S) test approach [54] is first applied to determine whether the distribution of fluxes of each biofilm-associated reactions (i.e., 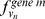 with *n*  = 1, 2, 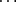, *num*_biofilm-reactions_), is of statistically significant change from the corresponding distribution obtained for the wild-type strain (i.e., 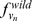, *n*  = 1, 2, 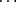, *num*_biofilm-reactions_). *num*_biofilm-reactions_ refers to the total number of biofilm-associated reactions. The two sample K-S test routine *kstest2* from MATLAB is used to perform K-S test, at a 5% significance level. If the two distributions 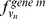 and 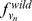 are different, 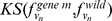 is equal to one, otherwise, 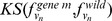 is set to zero.The difference between the two distributions 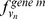 and 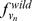 is further quantified by the following equation
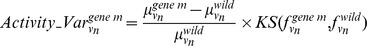
where 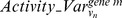 is the relative fold change of the activity (i.e., the flux) of the biofilm-associated reaction *n* upon the mutation of the essential gene *m*. Equation (2) is applied to all biofilm-associated reactions. An activity-variation vector is then constructed as shown in Equation (3) to describe the change in the distributions of fluxes across all biofilm-associated reactions upon the mutation of gene *m*.
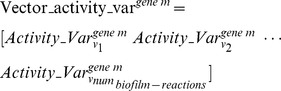
where 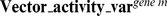 is referred to as the relative activity change profile for the gene *m* mutant in the following text. When Equation (3) is applied to all genes that are essential for planktonic growth, a matrix given by Equation (4) is constructed, which shows in each column the relative activity changes of one biofilm-associated reaction *i* for the mutants of all essential genes, and which shows in each row the relative activity changes in all biofilm-associated reactions upon the mutation of one essential gene.
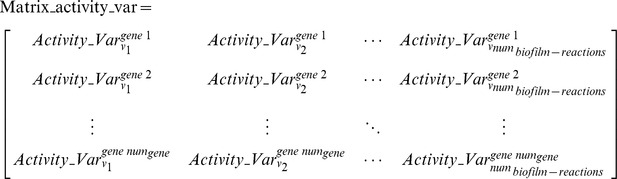
where *num_gene_* is the total number of essential genes for the planktonic pathogens. It can be seen from Equation (4) that the activity-variation vector 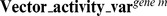 given in Equation (3) constitutes one row of **Matrix_activity_var**.
- Step 4: Potential gene targets are identified from the essential planktonic-growth genes by hierarchically categorizing them into different clusters based upon the capability of their mutants to form biofilms.One hypothesis to test in this work is that the mutation of an essential gene might induce the formation of biofilms. 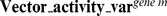 shown in Equation (3) describes the relative change of activity levels of all biofilm-associated reactions for the gene *m* mutant. 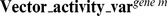 is thus used to represent the trend of biofilm formation upon the mutation of gene *m*. In particular, if certain elements in 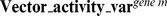, are of values larger than three, it means a significant amount of fluxes are re-distributed in the biofilm-associated reactions. The mutant thus exhibits high potential to switch from the planktonic state to the biofilm growth mode. In this work, the essential genes are categorized into different clusters based upon the capability of their mutants to form biofilms. The clustering result is then used to determine the gene targets to treat biofilm-associated pathogens. Specifically two relative activity change profiles are used to quantify the similarity of the impact of the mutation of two genes on biofilm formation via Equation (5).
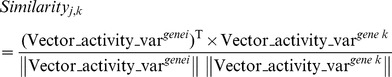


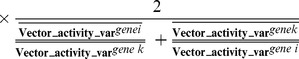
where *Similarity_i,k_* defines a similarity measure of the biofilm formation capability of the mutants of genes *i* and *k.* If the similarity measure is equal to unity, the mutations of the two genes have the same impact on the formation of biofilms. 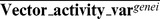 is the average relative activity change over all biofilm-associated reactions upon the mutation of gene *i*. The first term on the right-hand side of Equation (5) reflects the similarity of the shapes of activity change profiles contained in 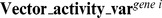 and 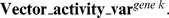
If these two activity change profiles are parallel, e.g., 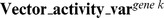 equal to 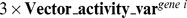, the first term is of a value of one. For this case, if only the first term is used as the measurement of similarity, the mutation of gene *k* is determined to have the same impact on biofilm formation as that of gene *i*. However, this is not true as the activity change magnitude in the mutant of gene *k* is three times of that for the gene *i* mutant. The second term on the right-hand side of Equation (5) is thus used to take the magnitude of activity change into account in the evaluation of similarity. If the activity change magnitudes of two profiles are quite different, the denominator of the second term is large. This reduces the similarity value of the two activity change profiles. Only two activity change profiles with similar shapes and comparable magnitudes are of a large similarity value.Based upon the quantified similarity, the genes essential for planktonic growth can be hierarchically categorized into different clusters via the hierarchical clustering routine *dendrogram* from MATLAB. Those essential planktonic-growth genes upon which the mutations lead to a low enhancement in the activity levels across biofilm-associated reactions are identified and regarded as good gene targets to treat biofilm-associated pathogens.
- Step 6: The metabolic reactions that facilitate biofilm formation in the single essential mutants are determined by identifying biofilm-associated reactions whose activity levels surge in most mutants. Each column in **Matrix_activity_var** shown in Equation (4) lists the relative change of the activity of one biofilm-associated reaction upon the mutation of all essential genes. Those biofilm-associated reactions whose activity levels significantly increase for most single-mutants account for the underlying metabolic mechanisms that facilitate biofilm formation in these mutants. In this work, the average relative activity change for a biofilm-associated reaction, 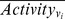, is quantified by Equation (6). Based upon the value of 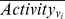 the biofilm-associated reactions are categorized into two types, one containing reactions with minor activity increase and the other containing reactions with large activity increase for most single-mutants. Two types were preferred here, as the simulation result showed that the relative flux activity changes of a biofilm-associated reaction over most mutants can be generally characterized by either minor changes or significant ones. The *k*-mean clustering routine in MATLAB is used to perform the clustering operation.
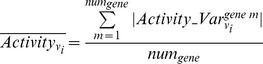

## Supporting Information

Table S1 The distribution of the essential genes from Cluster 1 of Figure 2 in biological subsystems.
(DOCX)
