# Supplementary material for: A Systems-Level Approach for Investigating Pseudomonas aeruginosa Biofilm Formation
Source: PLoS One. 2013 Feb 22;8(2):e57050. doi: 10.1371/journal.pone.0057050 (PMC3579789; doi:10.1371/journal.pone.0057050)
Supplement: Table S1 — The distribution of the essential genes from Cluster 1 of Figure 2 in biological subsystems. (DOCX) [file pone.0057050.s001.docx]

**Supplementary Table S1**

| Biological subsystems | Essential genes from Cluster 1 shown in Figure 2 |
| --- | --- |
| Vitamin and cofactor synthesis | PA0350(*folA*),PA0430(*metF*),PA0724(*coaA*),PA1004(*nadA*), PA1376(*aceK*),PA1758(*pabB*),PA2964(*pabC*),PA3088(*yfjB*), PA3111(*folC*),PA3666(*dapD*),PA4006(*nadD*),PA4053(*ribE*), PA4054(*ribB*),PA4524(*nadC*),PA4561(*ribF*),PA4655(*hemH*), PA4666(*hemA*),PA4750(*folP*),PA4920(*nadE*),PA5034(*hemE*), PA5243(*hemB*),PA5259(*hemD*),PA5260(*hemC*),PA4055(*ribC*), PA4056(*ribD*),PA0363(*coaD*),PA4397(*panE*),PA4529(*coaE*), PA4729(*panB*),PA4730(*panC*),PA5320(*coaC*),PA0582(*folB*), PA3296(*phoA*), PA1796(*folD*) |
| Amino acid catabolism | PA0025*(aroE),*PA0546(*metK*),PA0654(*speD*),PA0761(*nadB*), PA1162(*dapE*),PA1393(*cysC*),PA1681(*aroC*),PA1687(*speE*), PA3164(*pseudogene*),PA3763(*purL*),PA4442(*cysN*),PA4443(*cysD*), PA4759(*dapB*),PA5175(*cysQ*),PA5278(*dapF*),PA3659(*dapC*), PA5038(*aroB*),PA5039(*aroK*) |
| Cell wall synthesis | PA1959(*bacA*),PA2977(*murB*),PA2979(*kdsB*),PA3337(*rfaD*), PA4201(*ddlA*),PA4410(*ddlB*),PA4411(*murC*),PA4412(*murG*), PA4414(*murD*),PA4415(*mraY*),PA4416(*murF*),PA4417(*murE*), PA4425(*yraO*),PA4450(*murA*),PA4662(*murI*),PA4749(*glmM*), PA4996(*rfaE*),PA5161(*rmlB*),PA5162(*rmlD*),PA5163(*rmlA*), PA5164(*rmlC*),PA5549(*glmS*),PA4458(*yrbI*),PA3636(*kdsA*), PA0006(*yaeD*),PA5552(*glmU*) |
| Central metabolism | PA0330*(rpiA),*PA0548(*tktA*),PA2165(*glgA*),PA4457(*KdsD*), PA4748(*tpiA*),PA1614(*gpsA*),PA4670(*prs*),PA2053(*cynT*), |
| Transport | PA0280(*cysA*),PA0281(*cysW*),PA0283(*sbp*),PA1493(*cysP*), PA0282(*cysT*), |
| Nucleotide synthesis | PA1013(*purC*),PA3637(*pyrG*),PA4855(*purD*),PA0945(*purM*), PA3108(*purF*), PA2629(*purB*),PA4938(*purA*),PA4854(*purH*) |
| Nucleotide salvage | PA0342(*thyA*),PA2962(*tmk*),PA3163(*cmk*),PA3654(*pyrH*), PA3807(*ndk*),PA5336(*gmk*) |
| Lipid synthesis | PA0005*(lptA),*PA1806(*fabI*),PA2967(*fabG*),PA2968(*fabD*), PA2981(*lpxK*),PA3112(*accD*),PA3639(*accA*),PA3643(*lpxB*), PA3644(*lpxA*),PA3646(*lpxD*),PA3651(*cdsA*),PA3673(*plsB*), PA4050(*pgpA*),PA4406(*lpxC*),PA4693(*pssA*),PA4847(*accB*), PA4848(*accC*),PA4957(*psd*),PA4988(*waaA*),PA5008(*waaX*), PA5009(*waaP*),PA5010(*waaG*),PA5011(*waaC*),PA5012(*waaF*), PA2584(*pgsA*),PA1609(*fabB*) |
